# Genome-wide analysis of aberrantly expressed lncRNAs and miRNAs with associated co-expression and ceRNA networks in β-thalassemia and hereditary persistence of fetal hemoglobin

**DOI:** 10.18632/oncotarget.18263

**Published:** 2017-05-29

**Authors:** Ketong Lai, Siyuan Jia, Shanjuan Yu, Jianming Luo, Yunyan He

**Affiliations:** ^1^ Department of Pediatrics, The First Affiliated Hospital of Guangxi Medical University, Guangxi Zhuang Autonomous Region, Nanning 530021, China; ^2^ Guangxi Key Laboratory of Thalassemia Research, Guangxi Zhuang Autonomous Region, Nanning 530021, China

**Keywords:** lncRNA, miRNA, ceRNA, β-thalassemia, hereditary persistence of fetal hemoglobin

## Abstract

The implications of lncRNAs regarding fetal hemoglobin (HbF) induction in hemoglobin disorders remain poorly understood. In this study, microarray analysis was performed to profile lncRNAs, miRNAs and mRNAs in individuals with hereditary persistence of fetal hemoglobin (HPFH), β-thalassemia carriers with high HbF levels and healthy controls. The results show aberrant expression of 862 lncRNAs, 568 mRNAs and 63 miRNAs in the high-HbF group compared with the control group. Altered NR_001589, NR_120526, T315543, miR-486-3p, miR-19b-1-5p and miR-20a-3p expression was confirmed by quantitative reverse transcription-polymerase chain reaction, and Spearman correlation coefficients revealed significant positive correlations with HbF. Gene Ontology and Kyoto Encyclopedia of Genes and Genomes pathway enrichment analyses showed the hematopoietic cell lineage and apoptosis to be most significantly dysregulated in HbF induction. We analyzed coding genes near the lncRNAs and constructed a coding-noncoding co-expression network. Based on the results, lncRNAs likely contribute to increased HbF levels by activating expression of HBE1 and hematopoietic cell lineage-inducible molecules and by inhibiting that of apoptosis-inducible molecules. Finally, through construction of a competing endogenous RNA network, we found that 6 lncRNAs could bind competitively with miR-486-3p, resulting in increased HbF levels. Taken together, our findings provide new insights into the mechanisms of HbF induction and potentially provide new targets for the treatment of β-thalassemia major.

## INTRODUCTION

β-Thalassemia, one of the most common genetic disorders worldwide, is endemic in many tropical and sub-tropical areas, such as Mediterranean countries, the Middle East, North African, the Indian subcontinent and Southeast Asia [1]. Southern China also has a high prevalence of thalassemia, particularly in the Guangxi Zhuang Autonomous Region, where the total heterozygote frequency of thalassemia is 24.51% [2]. β-Thalassemia is caused by mutations, small deletions or insertions of one or two nucleotides in the β-globin genes. β-Thalassemia minor, which is caused by deficiency in one β-globin gene, can be asymptomatic or cause mild anemia. In contrast, homozygotes or double heterozygotes for β-thalassemia present severe diseases, including thalassemia major and thalassemia intermedia; individuals with the latter have milder symptoms and do not require regular transfusions [3]. In addition, β-thalassemia major patients appear healthy at birth because fetal hemoglobin (HbF, α2γ2) is still functioning. However, when the γ-globin gene is switched off at approximately the age of 4-6 months and there is little or no β-globin expression, severely progressive anemia occurs. These patients require lifelong transfusions and iron chelation therapy at regular intervals for survival [4]; if not regularly treated with transfusion, the majority of β-thalassemia major patients will die by 5 years of age [5].

HbF is the most abundant type of hemoglobin (Hb) in the fetus, but its level declines after birth and is largely replaced by adult Hb (HbA, α2β2). HbF comprises less than 5% of the total Hb at 6 months after birth and continues to decline, reaching an adult level of less than 1% by 2 years of age [6]. However, HbF remains at high levels in some adults due to pathological states of β-thalassemia major, thalassemia intermedia or some cases of β-thalassemia minor. HbF is also elevated under non-pathological conditions due to hereditary persistence of fetal hemoglobin (HPFH). HPFH is characterized by large, variable deletions of the human β-like globin cluster, which result in a variable compensatory increase in γ-globin chains [7]. β-Thalassemia patients with high HbF or HPFH have milder symptoms, and many do not require transfusions [8, 9]. Therapeutic approaches that increase the HbF concentration have great potential for ameliorating the clinical and hematologic severity of β-thalassemia major [10]. However, the molecular mechanisms of HbF induction in β-thalassemia remain obscure. Therefore, a better understanding of the relevant mechanism is critical for improved treatment of β-thalassemia major. Additionally, there is an urgent need to identify therapeutic targets focused on HbF.

Approximately 10-20% of transcripts encode proteins (mRNAs) and 80%-90% of transcripts are non-protein-coding RNAs that are not translated. These include microRNAs (miRNAs) and long noncoding RNAs (lncRNAs). miRNAs are ∼22 nucleotides in length [11], have crucial functions in the production and maturation of erythrocytes and also regulate expression of globin genes through post-transcriptional gene silencing [12]. The suppressive effects of several miRNAs (miR-15a/-16-1/-486-3p) on transcription factors (MYB and BCL11A) during β-globin gene expression may reactivate γ-globin gene expression and HbF synthesis [13, 14]. Regardless, no studies to date have analyzed expression of miRNAs in HbF induction by comparing β-thalassemia carriers with high HbF and normal controls. Further evaluation of aberrantly expressed miRNAs in HPFH and β-thalassemia minor with high HbF may reveal miRNA-mediated HbF induction pathways.

lncRNAs, a class of non-coding RNAs longer than 200 nucleotides, have become a focus of study in recent years. Increasing evidence shows that lncRNAs participate in various biological processes, such as X-chromosome inactivation, genomic imprinting, cell differentiation, and cellular developmental processes. It has also been suggested that lncRNAs have important functions in hematopoiesis and the pathogenesis of blood diseases [15–18]. However, no studies have focused on the involvement of lncRNAs in hemoglobin disorders and their effects on HbF induction.

In the present study, we investigated molecular mechanisms of HbF induction by evaluating expression of lncRNAs, mRNAs and miRNAs in circulating reticulocytes isolated from individuals with HPFH or β-thalassemia minor with high HbF. We analyzed Gene Ontology (GO) terms, and Kyoto Encyclopedia of Genes and Genomes (KEGG) pathways and coding genes near lncRNAs, and we constructed coding-noncoding co-expression (CNC) and competing endogenous RNA (ceRNA) networks to predict lncRNA function. Furthermore, we analyzed changes in lncRNA/miRNA expression associated with HbF levels. Our findings offer new insights into the mechanism of HbF induction and potentially provide new targets for increasing HbF in patients with β-thalassemia major and other hemoglobinopathies.

## RESULTS

### Microarray expression profiles of lncRNAs, mRNAs and miRNAs in reticulocytes

**Figure 1:**
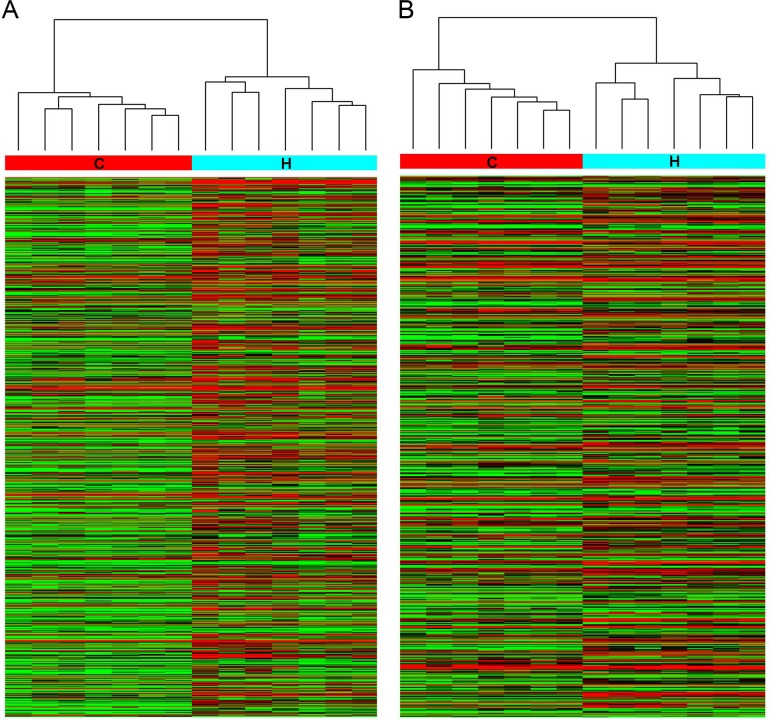
Hierarchical clustering of **(A)** lncRNA and **(B)** mRNA differential expression profiles between the high-HbF group and control group in 14 reticulocyte samples. The heat maps are based on expression values of significantly differentially expressed lncRNAs and mRNAs (absolute fold change ≥ 2.0 and P < 0.05) detected by microarray probes. “Red” and “Green” indicate expression above and below, respectively, relative expression. H: high-HbF group; C: control group.

**Figure 2:**
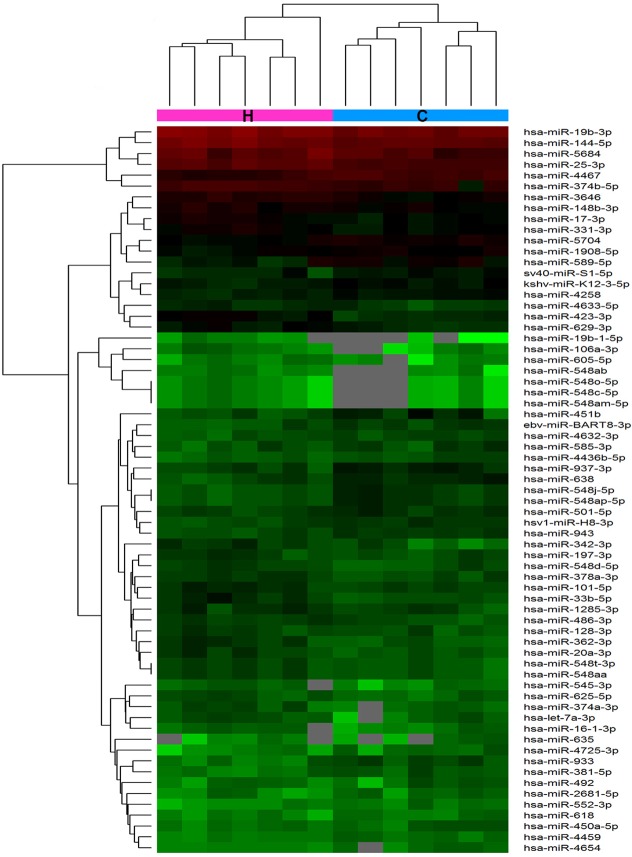
Hierarchical clustering of differentially expressed miRNA between the high-HbF group and control group in 14 reticulocyte samples The heat maps are based on expression values of significantly differentially expressed miRNAs (absolute fold change ≥ 1.5 and P < 0.05) detected by microarray probes. “Red” and “Green” indicate expression above and below, respectively, relative expression.

To explore the potential biological functions of lncRNAs in HbF induction, we examined the expression patterns of lncRNAs, mRNAs and miRNAs in reticulocytes from individuals with HPFH and β-thalassemia minor with high HbF and in age- and gender-matched controls with normal HbF levels. In total, 862 lncRNAs and 568 mRNAs showed a ≥ 2.0-fold change (P < 0.05) in 7 paired samples (Figure 1). Of these, 605 lncRNAs were up-regulated, and 257 were down-regulated; 324 mRNAs were up-regulated, and 244 were down-regulated miRNA microarray assay data indicated a fold change ≥ 1.5 between the high-HbF and control groups for 63 miRNAs (P < 0.05), including 34 up-regulated and 29 down-regulated miRNAs (Figure 2).

### Validation of dysregulated lncRNAs and miRNAs

**Figure 3:**
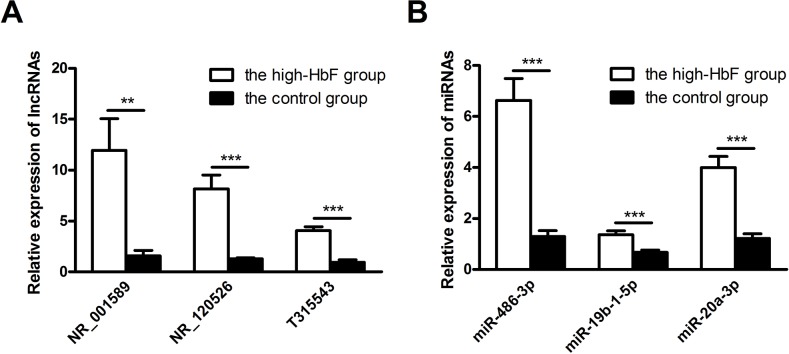
Validation of microarray data by qRT-PCR **(A)** Three up-regulated lncRNAs and **(B)** three up-regulated miRNAs were validated by qRT-PCR using RNA extracted from reticulocytes of 13 subjects with HPFH and β-thalassemia minor with high HbF and 13 controls. The relative expression level of each RNA was normalized, and the data displayed in histograms are expressed as the means ± SD, **P<0.01; ***P<0.001 comparing high-HbF and normal-HbF subjects.

To confirm the microarray data, we selected 3 lncRNAs and miRNAs each for quantitative reverse transcription-polymerase chain reaction (qRT-PCR) verification in 13 pairs of samples. NR_001589 (located upstream of β-globin locus), NR_120526 (located on chromosome 11), and T315543 (located on chromosome 6) with greater fold changes as well as miR-486-3p, miR-19b-1-5p and miR-20a-3p, also found to be aberrantly expressed in other studies of HbF induction, were selected for this assay. Based on the results, lncRNAs NR_001589, NR_120526 and T315543 were all up-regulated (Figure 3A). Additionally, miR-486-3p, miR-19b-1-5p and miR-20a-3p were up-regulated in the high-HbF group compared with the controls (Figure 3B). The results were consistent with those of the microarray assay.

### Assessment of associations between lncRNAs/miRNAs and HbF levels

**Figure 4:**
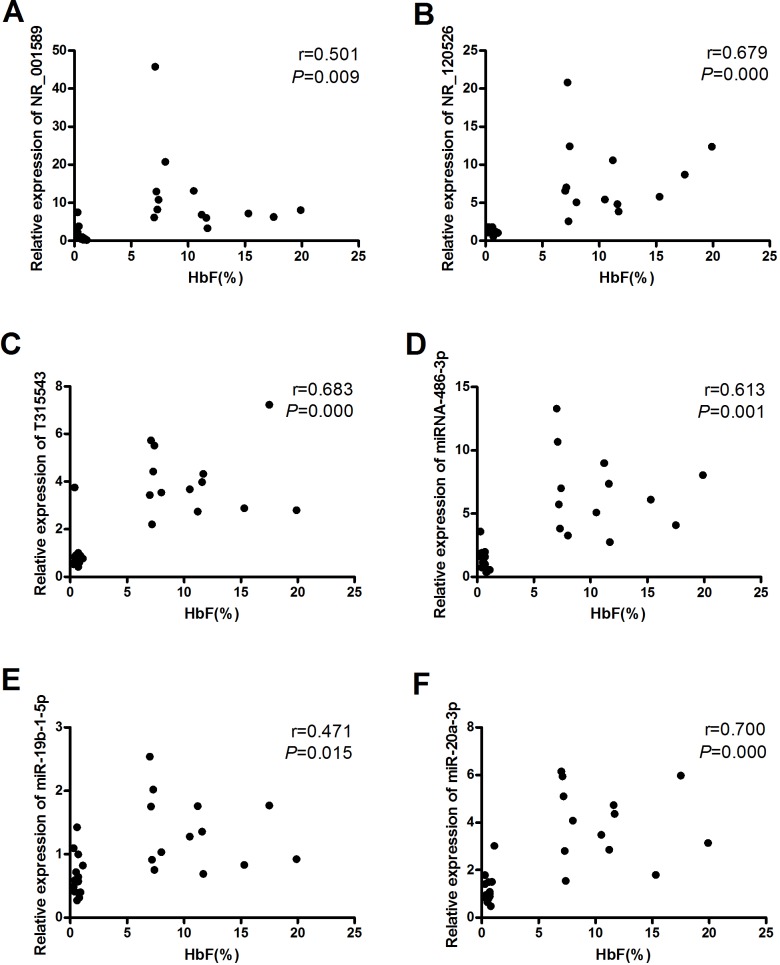
Associations between lncRNAs/miRNAs and HbF levels The Spearman correlation coefficient was utilized to evaluate associations between **(A)** NR_001589 and HbF, **(B)** NR_120526 and HbF, **(C)** T315543 and HbF, **(D)** miR-486-3p and HbF, **(E)** miR-19b-1-5p and HbF and **(F)** miR-20a-3p and HbF.

The Spearman correlation coefficient was used to evaluate associations between the levels of lncRNAs and miRNAs verified by qRT-PCR and HbF levels. We observed significant positive correlations between NR_001589 and HbF (r = 0.501, P = 0.009), NR_120526 and HbF (r = 0.679, P = 0.000), T315543 and HbF (r = 0.683, P = 0.000), miR-486-3p and HbF (r = 0.613, P = 0.001), miR-19b-1-5p and HbF (r = 0.471, P = 0.015) and miR-20a-3p and HbF (r = 0.700, P = 0.000) (Figure 4A-4F).

### Coding genes near lncRNAs

lncRNAs have been classified into different subgroups based on the properties of their genetic loci. For example, some lncRNAs are located within intergenic spaces, i.e., between coding genes (large intergenic non-coding RNAs or lincRNAs). Through chromosomal localization and BLAST sequence alignment, we analyzed lincRNAs and their associated protein-coding genes (distance < 300 kb) to reveal the functions these of lincRNAs. The results show aberrant expression of 56 lincRNAs (fold change ≥ 2.0, P < 0.05) in the high-HbF group compared with the control group. Of these, 45 were up-regulated and 11 down-regulated. In addition, we identified some nearby coding genes that may be regulated by these lincRNAs (Supplementary Table 1).

### GO and KEGG pathway analyses

**Figure 5:**
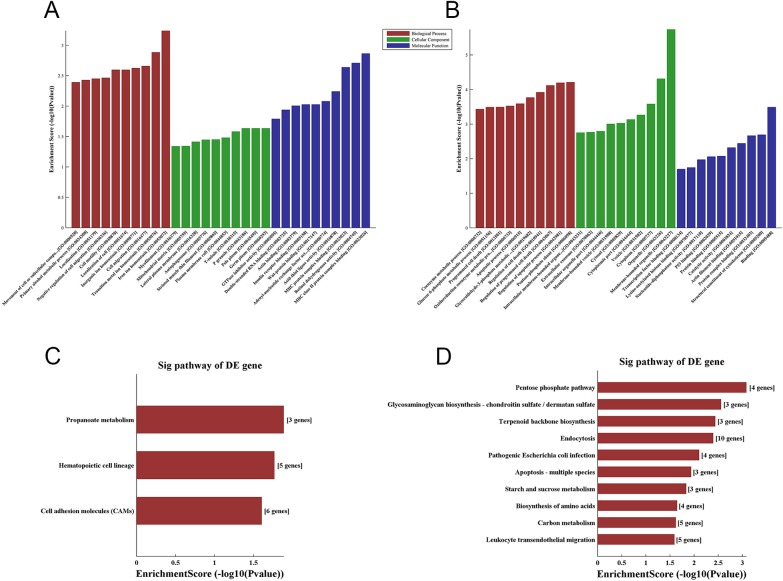
GO and KEGG pathway analysis of differentially expressed mRNAs GO annotation of **(A)** up-regulated and **(B)** down-regulated mRNAs, with the top ten enrichment score-covering domains of biological processes, cellular components and molecular functions. **(C)** The three significant pathways of up-regulated mRNAs. **(D)** The 10 significant pathways of down-regulated mRNAs. Enrichment score values were calculated as -log 10(p values).

To explore the potential functions of lncRNAs in HbF induction, we performed GO analysis on aberrantly expressed mRNAs. The GO project mainly covers three domains (Biological Process, Cellular Component and Molecular Function) and provides a controlled vocabulary to describe gene and gene product attributes in a given organism (http://www.geneontology.org). In our study, up-regulated mRNAs were found to be involved in such biological processes of ion homeostasis, transition metal ion homeostasis and cellular migration (Figure 5A), whereas down-regulated mRNAs, such as GATA1, BECN2, BIRC6 and HTRA2, were most relevant to regulation of apoptotic process, cell death and programmed cell death (Figure 5B). The significant GO terms for the cellular component and molecular function categories are shown in Figure 5A and Figure 5B.

To investigate the pathways and molecular interactions of the identified genes, we used the latest version of the KEGG database for pathway enrichment analysis to examine aberrantly expressed mRNAs (http://www.genome.jp/kegg). The results demonstrated enrichment among the up-regulated genes for 3 pathways, propanoate metabolism, hematopoietic cell lineage and cell adhesion molecules (Figure 5C); down-regulated genes were enriched for the pentose phosphate pathway, glycosaminoglycan biosynthesis, terpenoid backbone biosynthesis and apoptosis (Figure 5D). Five significantly up-regulated genes in the hematopoietic cell lineage pathway were highlighted, including TFRC, CSF2, CSF3, HLA-DOA and MS4A1. Three significantly down-regulated genes involved in apoptosis, BECN2, BIRC6 and HTRA2, were also found. The pathways of significantly differentially expressed genes in the different groups are shown in Figure 5.

### lncRNA-mRNA co-expression network

**Figure 6:**
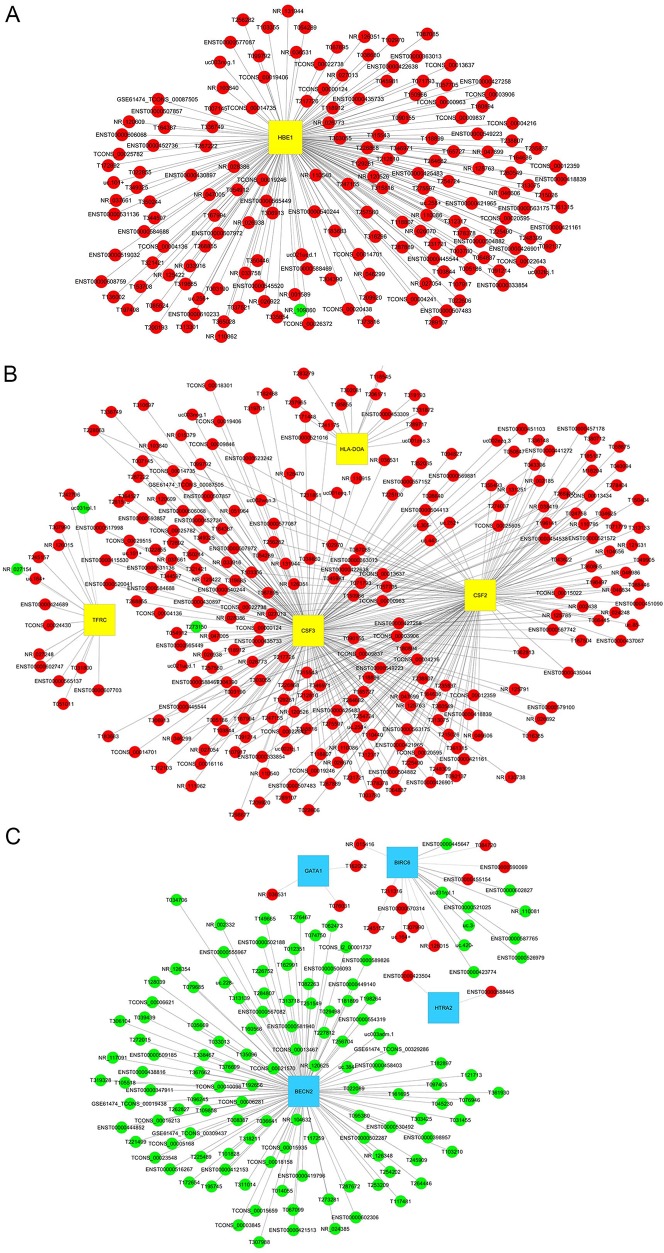
lncRNA-mRNA co-expression network The network is based on Pearson correlation coefficient (the absolute value of PCC ≥ 0.90 and P-value < 0.05). **(A)** A total of 167 lncRNAs interact with HBE1. **(B)** A total of 265 lncRNAs interact with 4 mRNAs in the hematopoietic cell lineage pathway. **(C)** A total of 140 lncRNAs interact with 4 mRNAs associated with regulation of apoptosis. Yellow squares indicate up-regulated mRNAs and blue squares down-regulated mRNAs. Red nodes indicate up-regulated lncRNAs and green nodes down-regulated lncRNAs.

To characterize the functions of lncRNAs, 10 differentially expressed mRNAs were selected to construct a co-expression network to according to the degree of correlation (Figure 6). The selected mRNAs were GATA1, BECN2, BIRC6 and HTRA2, implicated in the regulation of apoptosis, and TFRC, CSF2, CSF3, HLA-DOA and MS4A1, implicated in the hematopoietic cell lineage and HBE1. The network showed that HBE1 was correlated with 167 lncRNAs (Figure 6A), that TFRC, CSF2, CSF3 and HLA-DOA were correlated with 265 lncRNAs (Figure 6B), and that GATA1, BECN2, BIRC6 and HTRA2 were correlated with 140 lncRNAs (Figure 6C). No correlations were observed for MS4A1.

### Construction of a ceRNA network

**Figure 7:**
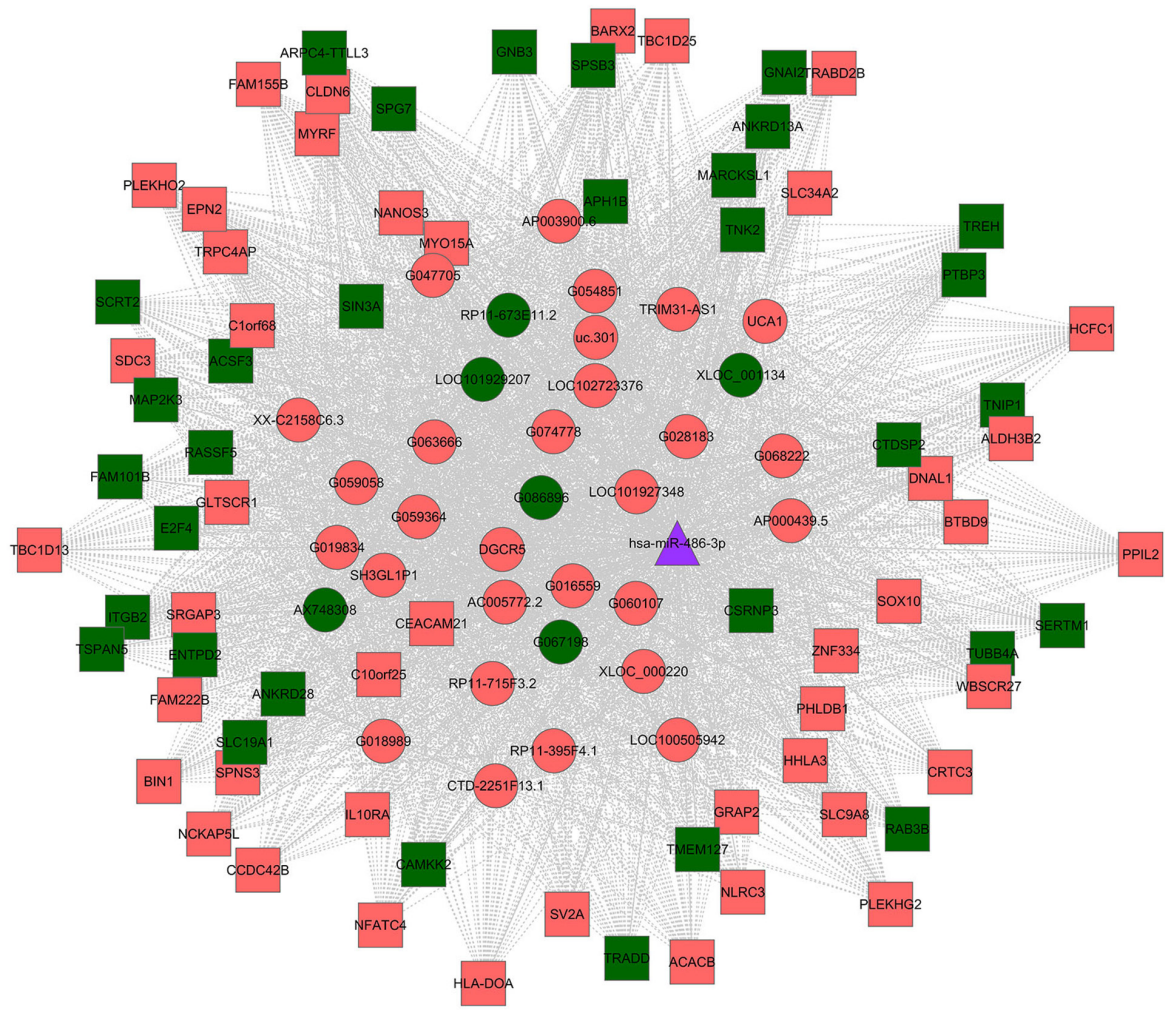
Differentially expressed lncRNA-mediated ceRNA network in β-thalassemia and HPFH The lncRNA-miR-486-3p-mRNA network for β-thalassemia and HPFH is shown. Red nodes indicate an increased level of expression, while green nodes indicate a decreased level of expression. lncRNAs and mRNAs are indicated as round and square symbols, respectively.

According to the ceRNA hypothesis, competing ceRNAs, including some lncRNAs, can compete for the same miRNA response elements (MREs) to suppress miRNA function. To reveal how specific miRNAs interact with lncRNAs and coding genes, we constructed a lncRNA-miRNA-mRNA ceRNA network for HPFH and β-thalassemia carriers with high HbF using our microarray data. We selected one differentially expressed miRNA (miR-486-3p) that contains a common MRE-based binding site. The ceRNA network showed that 34 lncRNAs (28 up-regulated and 6 down-regulated) had the same MREs for miR-486-3p (Figure 7). The downed-regulated lncRNAs might be ceRNAs for miR-486-3p targeting mRNAs, such as SIN3A, APH1B and CSRNP3. These RNA interactions provide novel insight into the mechanisms of HbF induction in β-thalassemia.

## DISCUSSION

Noncoding RNAs have important regulatory functions in gene expression and biological processes, including cell division, survival, and differentiation. Although the functional relevance of many miRNAs has been proven in normal and malignant hematopoiesis, the involvement of lncRNA in hematopoiesis is only beginning to be defined. The lncRNA lincRNA-EPS is essential for the maturation of red blood cells, and it can repress PYCARD, a proapoptotic gene, to promote the survival of mouse erythroblasts [19]. RNA-seq was used to define lncRNAs expressed during murine erythropoiesis, revealing 12 lncRNAs with potential functions [20]. As a precursor of miR-675, imprinting of the lncRNA H19 controls mouse hematopoietic stem cell quiescence and is highly associated with chronic myeloid leukemia, chronic myelomonocytic leukemia, and acute myelogenous leukemia [21–23]. However, comprehensive analysis of differentially expressed profiles of lncRNAs in β-thalassemia has not yet been reported. Thus, to evaluate the functions of lncRNAs in HbF induction, we applied microarray to explore genome-wide expression profiles of lncRNAs and mRNAs in 7 subjects with HPFH or β-thalassemia minor with high HbF and 7 matched subjects with normal HbF

To the best of our knowledge, this is the first study to examine differentially expressed lncRNAs in β-thalassemia and HPFH. The microarray results show 635, 34 and 273 up-regulated and 205, 29 and 159 down-regulated lncRNAs, miRNAs and mRNAs, respectively, between the high-HbF and normal HbF samples. Moreover, 3 dysregulated lncRNAs and miRNAs each were verified by qRT-PCR. Our further bioinformatic prediction analysis revealed a set of lncRNAs as potential regulators of gene expression in HbF induction. These data will allow for intriguing areas of inquiry for more in-depth exploration.

The differentially expressed lncRNAs identified are widely distributed throughout the genome, including chromosomes 6 and 11. The γ- and β-globin genes are located on chromosome 11. In addition, some quantitative trait locis (QTLs) that may be regulatory regions accounting for the persistence of γ-globin gene expression have been found on chromosomes 6 and 11, such as the chromosome 6q23.3 HBS1L-MYB and β-globin loci [24, 25]. In particular, NR_001589, with 9.63-fold up-regulation in the high-HbF group, is of interest because it is located upstream of the β-globin locus. Furthermore, the adjacent protein-coding gene results showed that NR_001589 was associated with HBE1, up-regulated 62-fold in microarray assays. The human β-globin locus contains five functional genes in the order 5′-ε-Gγ-Aγ-δ-β-3′. HBE1 encodes ε-globin and is normally expressed in the embryonic yolk sac. 2 ε-globin chains together with 2 ζ-globin/α-globin chains constitute embryonic Hb Gower I/ Hb Gower II, which is normally supplanted by HbF during the early gestational period [26]. Our results suggest that embryonic ε-globin gene expression is preserved in HPFH and β-thalassemia carriers with high HbF and NR_001589 may be involved in activating HBE1.

Although differentially expressed lncRNAs are thought to control biological processes, most have not yet been studied, and new types of gene regulators are only now being discovered. Accordingly, we performed GO analysis to predict the potential functions of these dysregulated lncRNAs. We mainly identified mRNAs that regulate several biological processes: the top terms were regulation of apoptotic process, cell death and programmed cell death, with GATA1, BECN2, BIRC6 and HTRA2 enrichment. Furthermore, pathway analysis was performed to gain insight into the biological pathways potentially involved in HbF induction. In our study, both GO and pathway analyses suggested the involvement of apoptosis in HbF induction. GATA1 is an important hematopoietic transcription factor in erythrocyte production. During the embryonic period, GATA1 has a prominent role in the last stages of erythropoiesis by regulating genes involved in cell division and apoptosis leading to terminal maturation; by forming a protein complex and binding to γ-globin promoters, it also has a function in suppressing the adult γ-globin gene [27]. Our results indicate that down-regulated GATA1 may inhibit reticulocyte apoptosis and increase HbF levels. Our pathway analysis also revealed the hematopoietic cell lineage as another important pathway critical to hematopoiesis. Significant up-regulation of transcription was found for five genes in the pathway: TFRC, CSF2, CSF3, HLA-DOA and MS4A1. The transferrin receptor TFRC (CD71) has an important function in the process of iron absorption and the terminal differentiation of erythroid cells. It has been reported that during erythroid differentiation, aberrant expression of TFRC, in part affected by miR-210, leads to increased levels of α- and γ-globin followed by HbF [28]. In our present study, levels of GATA1, BECN2, BIRC6, HTRA2, TFRC, CSF2, CSF3, HLA-DOA and MS4A1 expression, involved in apoptosis and the hematopoietic cell lineage, were all significantly altered, suggesting that these aberrantly expressed transcripts may be highly associated with HbF induction.

To further explore lncRNA functions, CNC network analysis was performed based on 862 lncRNAs and 10 mRNAs differentially expressed between healthy controls and individuals with HPFH or β-thalassemia minor with high HbF. The 10 differentially expressed mRNAs were HBE1 and those implicated in regulation of apoptosis and the hematopoietic cell lineage. The network showed that 167 lncRNAs interacted with HBE1, 265 lncRNAs with TFRC, CSF2, CSF3 and HLA-DOA, involved in the hematopoietic cell lineage pathway, and 140 lncRNAs with GATA1, BECN2, BIRC6 and HTRA2, associated with regulation of apoptosis. The network also indicated that NR_001589 is a potential regulator of HBE1 and that NR_120526 and T315543 are potential regulators of CSF2 and CSF3. Together with the finding of positive and significant correlations between NR_001589, NR_120526 and T315543 expression and HbF levels, our results strongly indicate that NR_001589, NR_120526 and T315543, as genetic modifiers of HbF induction, are potential therapeutic targets for increasing HbF levels. Although further investigation is necessary to confirm the results, our study suggests that lncRNAs likely contribute to increasing HbF levels by activating the embryonic ε-globin gene HBE1, by stimulating expression of hematopoietic cell lineage-inducible molecules and by inhibiting expression of apoptosis-inducible molecules.

Previous studies have shown that lncRNAs can serve as natural miRNA sponges to suppress miRNAs and prevent them from binding to mRNAs, causing suppression of miRNA function [29]. Therefore, identifying well-established miRNAs that bind to lncRNAs may help in elucidating the function of lncRNAs. Many studies have analyzed expression of miRNAs in HbF induction by comparing miRNA expression in adult peripheral blood samples and cord blood samples and in patients with sickle-cell disease or trisomy 13 cases with high HbF and normal controls [13, 30–32]. However, no study to date has focused on HPFH or β-thalassemia carriers with high HbF and normal controls of examined miRNAs together with lncRNAs. Here, we report altered expression of several specific lncRNAs and miRNAs in reticulocytes of individuals with HPFH and β-thalassemia minor with high HbF. Some intersections of miRNAs, such as miR-486-3p, miR-19b-1-5p and miR-20a-3p, were found by comparing with other studies of HbF induction. These intersections strongly suggest their contributions to hematopoiesis and hemoglobin diseases for HbF induction. miR-486-3p targets and suppresses the transcription factor BCL11A by binding to the extra-long isoform of the BCL11A 3′UTR to increase γ-globin expression [14]. We constructed an lncRNA-miR-486-3p-mRNA ceRNA network based on our microarray data. The ceRNA network showed that 34 lncRNAs had the same MREs for miR-486-3p. miR-486-3p, which was significantly up-regulated in the high-HbF group, was negatively correlated with 6 down-regulated lncRNAs. According to the ceRNA hypothesis, the 6 down-regulated lncRNAs may control expression of coding genes by sponging miR-486-3p and possess regulatory functions as ceRNAs, resulting in increased HbF levels. The pioneering discovery might enrich our understanding of HbF induction in HPFH and β-thalassemia and warrants further study.

This prospective translational study identified RNAs in reticulocytes and provides initial evidence for HbF induction in HPFH and β-thalassemia. Nonetheless, the findings should be evaluated with regard to study limitations. First, by analyzing late-stage erythroblasts instead of erythroid progenitors from the bone marrow, some molecular changes may have been missed. In addition, the target genes of the identified aberrantly expressed lncRNAs and miRNAs were predicted using a bioinformatic approach, and confirmation should be obtained by biological function analysis. Regardless, initial associations that can be used as hypothesis-generating findings for future experiments are provided.

Taken together, we found a profile of dysregulated lncRNAs and miRNAs associated with HbF induction in HPFH and β-thalassemia carriers. These data lay a foundation for further functional research on lncRNAs in Hb disorders. lncRNAs likely contribute to increased HbF levels by activating expression of HBE1 and hematopoietic cell lineage-inducible molecules and by inhibiting expression of apoptosis-inducible molecules. NR_001589, NR_120526 and T315543, associated with HbF levels, may serve as therapeutic targets for increasing HbF levels in patients with β-thalassemia major and other hemoglobinopathies. The observed significant associations of lncRNAs and miRNAs with HbF reinforce the need for future studies focusing on lncRNA-mediated mechanisms in HbF induction.

## MATERIALS AND METHODS

### Subjects and clinical characteristics

Human study protocols were approved by the medical ethics committee of the First Affiliated Hospital of Guangxi Medical University. All participants provided informed consent before commencement of the study. Diagnosis was carried out by Hb electrophoresis, high-performance liquid chromatography (HPLC), and gene analysis for thalassemia [2]. Subjects with HPFH or β-thalassemia minor with high HbF (>5%) were enrolled into the high-HbF group (H); age- and gender-matched subjects with normal HbF (<3.3%) were recruited into the control group (C). In total, the high-HbF group consisted of 20 subjects and the control group 20 healthy subjects. All subjects were residents of the Guangxi Region. None of subjects had hematological malignancies or a history of blood transfusions. Most of the subjects were female (80.0%) and of Zhuang ethnicity (65.0%). The high-HbF group and the control group had similar age, gender and ethnicity distributions (Table 1). Of the 20 subjects in the high-HbF group, 7 were selected for the microarray assay, including 4 with β-thalassemia minor and 3 with HPFH; 13 subjects were used for qRT-PCR validation, including 9 with β-thalassemia minor and 4 with HPFH.

**Table 1:** The characteristics of studied subjects

Items		The high-HbF group	The control group
Number		20	20
HbF (mean ± SD)		10.66±3.59%	0.54±0.23%
Age (mean ± SD)		30.80±4.23	30.15±4.20
Gender	Female	16	16
	Male	4	4
Ethnicity	Zhuang	13	12
	Han	7	8

### Isolation of nucleated red blood cells (NRBCs) and reticulocytes

Two milliliters of peripheral blood anticoagulated with EDTA was collected from each subject and centrifuged at 500x *g* for 2 minutes. The plasma supernatant containing platelets was removed. Later, each sample was depleted of CD45+ cells to remove leukocytes, and then NRBCs and reticulocytes were enriched through positive selection using anti-CD71 MicroBeads (Miltenyi Biotec, Germany). The purity of CD45^−^ and CD71^+^ cells isolated was determined using fluorescein isothiocyanate (FITC)-labeled anti-CD71 and allophycocyanin (APC)-labeled anti-CD45 antibodies (BD Biosciences, USA). The purified cells (< 0.8% CD45^+^ and > 90% CD71^+^) consisted primarily of reticulocytes and nucleated erythrocytes, all of which were used for RNA extraction.

### RNA extraction

Total RNA was extracted from reticulocytes using TRIzol (Invitrogen life technologies, USA) in accordance with the manufacturer's protocol. The quantity and quality of the total RNA were assessed using a NanoDrop ND-1000 spectrophotometer (NanoDrop, USA). Denaturing agarose gel electrophoresis was used to determine the integrity of the RNA.

### Microarray assay

Human LncRNA Microarray V4.0 (Arraystar, USA) is designed for global profiling of human lncRNAs and protein-coding transcripts. Approximately 40,173 lncRNAs and 20,730 coding transcripts can be detected by the fourth-generation lncRNA microarray. Microarray hybridization was performed based on the manufacturer's standard protocols (Agilent Technology, USA), including RNA purification, transcription into fluorescent cRNA, and cDNA hybridization onto Microarray V4.0. The hybridized arrays were washed, fixed and scanned using a Microarray Scanner (Agilent, USA). Agilent Feature Extraction software (version 11.0.1.1) was used to analyze the array images acquired. Quantile normalization and further data analysis were performed using the GeneSpring GX v12.1 software package (Agilent, USA).

The miRNA microarray assay included labeling, hybridization, scanning, normalization, and data analysis. The labeling kit was used according to the manufacturer's instructions (Exiqon, Denmark). The labeled samples were hybridized to the miRCURY LNA™ microRNA array (Exiqon, Denmark), which covers 3,557 miRNA. The arrays were washed and immediately scanned using a microarray scanner (Axon, USA). Finally, the scanned images were imported into GenePix software (Axon, USA) for grid alignment and data extraction.

### qRT-PCR

Thirteen paired-samples (13 subjects in the high-HbF group and 13 subjects in the control group) were used for qRT-PCR validation. Total RNA was reverse-transcribed into cDNA using SuperScriptTM III Reverse Transcriptase (Invitrogen, USA) and MMLV Reverse Transcriptase (Epicentre, USA) for lncRNAs and miRNAs, respectively, according to the manufacturer's instructions. In a reaction volume of 10 μl, the reverse transcription included 5 μl 2×Master Mix (Arraystar, USA), 0.5 μl PCR Forward Primer, 0.5 μl PCR Reverse Primer, 2 μl template cDNA, and 2 μl double distilled water. qRT-PCR was performed using ViiA 7 Real-Time PCR System(ABI, USA), with the following cycling conditions: 95°C for 10 min followed by 40 cycles of 95°C (10 sec) and 60°C (60 sec). The lncRNA and miRNA PCR results were quantified using the 2^−ΔΔct^ method, with normalization using β-actin and U6, respectively. The data represent the mean values of three experiments. The sequences of the qRT-PCR primersused are listed in Supplementary Table 2.

### Associations between lncRNAs/miRNAs and HbF levels

The Spearman correlation coefficient was used to evaluate associations between lncRNAs/miRNAs and HbF levels.

### GO and KEGG pathway analyses

GO analysis allows functional association of differentially expressed mRNAs using three structured networks of defined terms that describe gene product attributes. P-values denote the significance of GO term enrichment in the differentially expressed mRNAs. The P-value cut-off was set at 0.05. KEGG pathway analysis for significantly aberrantly expressed mRNAs was also performed. The P-value, which denotes the significance of the pathway, was set to a cut-off of 0.05.

### Analysis of the lncRNA-mRNA co-expression network

lncRNA-mRNA co-expression analysis was based on calculating the Pearson correlation coefficient (PCC) between coding genes and lncRNAs according to their specific expression levels. Absolute values of PCC ≥0.90 and P-value <0.05 were recommended and retained for further analysis.

### Construction of dysregulated lncRNA-associated ceRNA network

Those lncRNAs and mRNAs with expression levels sharing a meaningful correlation were subjected to further analysis. lncRNA-miRNA interactions were predicted using miRcode (http://www.mircode.org/), and miRNA-mRNA interactions were predicted using Targetscan (http://www.targetscan.org/). Overlap of the same miRNA seed sequence binding site on both lncRNAs and mRNAs predicted a lncRNA-miRNA-mRNA interaction.

### Statistical analysis

All statistical data were analyzed with SPSS 20.0 software (SPSS Inc., Chicago). Data are shown as the mean ± SD. Student's t-test was performed to analyze the statistical significance of the microarray and qRT-PCR results. Statistical differences were considered significant at P < 0.05.

## SUPPLEMENTARY MATERIALS TABLE





## Abbreviations

ceRNA: competing endogenous RNA
CNC network: coding-noncoding co-expression network
GO: Gene Ontology
Hb: hemoglobin
HbF: fetal hemoglobin
HPFH: hereditary persistence of fetal hemoglobin
HPLC: high-performance liquid chromatography
KEGG: Kyoto Encyclopedia of Genes and Genomes
lncRNAs: long noncoding RNAs
miRNAs: microRNAs
MREs: miRNA response elements
NRBCs: nucleated red blood cells
PCC: Pearson correlation coefficient
qRT-PCR: quantitative reverse transcription-polymerase chain reaction
QTLs: quantitative trait locis
