# Supplementary material for: Genome-wide analysis of aberrantly expressed lncRNAs and miRNAs with associated co-expression and ceRNA networks in β-thalassemia and hereditary persistence of fetal hemoglobin
Source: Oncotarget. 2017 May 29;8(30):49931–43. doi: 10.18632/oncotarget.18263 (PMC5564818; doi:10.18632/oncotarget.18263)
Supplement: Supplementary file 1 [file oncotarget-08-49931-s001.pdf]

Genome-wide analysis of aberrantly expressed lncRNAs and miRNAs with associated co-expression and ceRNA networks in β-thalassemia and hereditary persistence of fetal hemoglobin

SUPPLEMENTARY INFORMATION

Supplementary Table 1: Coding genes near lncRNAs

See Supplementary File 1

Supplementary Table 2: The sequences of the qRT-PCR primers

Primer name	Sequence (5′-3′)		Product length (bp)
β-actin (H)	Forward	GTGGCCGAGGACTTTGATTG	73
	Reverse	CCTGTAACAACGCATCTCATATT	
NR_001589	Forward	TTCAGCGAGGATTTTACCC	90
	Reverse	CTCAGTGGTCTTGTGGGCTA	
NR_120526	Forward	GAGGCAACTACAATCCAGAGGA	82
	Reverse	ACATAATGGGCAGAGTGAAAGAG	
T315543	Forward	GTTGGATGGTGAAGGAGAAGG	57
	Reverse	GTTTTGTTGCTAGAAGCAGAGGA	
U6	Forward	GCTTCGGCAGCACATATACTAAAAT	89
	Reverse	CGCTTCACGAATTTGCGTGTCAT	
miR-486-3p	Forward	GGAACGGGGCAGCTCAGTA	63
	Reverse	GTGCGTGTCGTGGAGTCG	
miR-19b-1-5p	Forward	GGCGAGTTTTGCAGGTTTG	65
	Reverse	GTGCGTGTCGTGGAGTCG	
miR-20a-3p	Forward	GGGGAACTGCATTATGAGCA	65
	Reverse	GTGCGTGTCGTGGAGTCG	
