# Supplementary material for: Genome-wide analysis of aberrantly expressed lncRNAs and miRNAs with associated co-expression and ceRNA networks in β-thalassemia and hereditary persistence of fetal hemoglobin
Source: Oncotarget. 2017 May 29;8(30):49931–43. doi: 10.18632/oncotarget.18263 (PMC5564818; doi:10.18632/oncotarget.18263)
Supplement: Supplementary file 2 [file oncotarget-08-49931-s002.docx]

**Supplementary Table 1: Coding genes near lncRNAs**

| **Seqname** | **P-value** | **Fold change** | **Regulation** | **Chromo-some** | **Genome relationship** | **Nearby gene** |
| --- | --- | --- | --- | --- | --- | --- |
| ENST00000418839 | 0.000213774 | 3.4949921 | Up | chr3 | Downstream | GHSR |
| ENST00000441272 | 0.000927849 | 4.2855315 | Up | chr1 | Upstream | HDGF |
| ENST00000445681 | 0.003123191 | 2.3654044 | Up | chr7 | Upstream | TPST1 |
| ENST00000455154 | 0.007037974 | 2.1906663 | Up | chr11 | Upstream | OSBPL5 |
| ENST00000518590 | 0.033577531 | 2.2177375 | Up | chr8 | Upstream | SLC25A37 |
| ENST00000521016 | 0.045328741 | 2.8610747 | Up | chr8 | Upstream | CSGALNACT1 |
| ENST00000526979 | 0.00274836 | 2.0157681 | Down | chr11 | Downstream | ARL2 |
| ENST00000562328 | 0.04177597 | 2.0611037 | Down | chr2 | Upstream | RUFY4 |
| ENST00000565449 | 0.00016213 | 4.6110039 | Up | chr7 | Upstream | WEE2 |
| ENST00000585471 | 0.041873206 | 2.097979 | Down | chr17 | Upstream | FMNL1 |
| ENST00000603265 | 0.003304756 | 2.1660918 | Up | chr21 | Upstream | BAGE3 |
| ENST00000609708 | 0.001138057 | 2.1838325 | Down | chr19 | Downstream | BRD4 |
| GSE61474_TCONS_00019438 | 0.005714489 | 2.6514371 | Down | chr1 | Downstream | CEP85 |
| GSE61474_TCONS_00260336 | 0.000112458 | 2.7369203 | Up | chr4 | Downstream | SAP30 |
| NR_001589 | 2.73108E-05 | 3.6481629 | Up | chr11 | Upstream | HBE1 |
| NR_002158 | 0.015891352 | 2.0610247 | Up | chr7 | Upstream | BAC05914.1 |
| NR_002185 | 0.000898988 | 6.3650671 | Up | chr2 | Upstream | ANKRD53 |
| NR_024397 | 0.024294451 | 2.0632551 | Down | chr10 | Upstream | NUTM2D |
| NR_024472 | 0.018453357 | 2.0062147 | Up | chr10 | Downstream | C10orf25 |
| NR_026564 | 0.003400881 | 3.0959544 | Up | chr11 | Upstream | MRGPRX3 |
| NR_026827 | 0.005456529 | 2.1291096 | Up | chr10 | Downstream | ZNF33B |
| NR_126384 | 0.003720471 | 2.3744131 | Up | chr2 | Downstream | ANKRD53 |
| T005283 | 0.019812066 | 10.7803084 | Up | chr1 | Upstream | EPHA8 |
| T008013 | 0.020063288 | 2.2301019 | Up | chr1 | Downstream | STK40 |
| T014840 | 0.002227586 | 3.5319365 | Up | chr1 | Upstream | ZNF326 |
| T022606 | 0.000127488 | 4.3188386 | Up | chr1 | Downstream | HDGF |
| T022855 | 5.61386E-05 | 5.2866372 | Up | chr1 | Downstream | FCRL4 |
| T052660 | 0.012838321 | 2.8902793 | Up | chr11 | Downstream | TOLLIP |
| T058875 | 0.005916152 | 2.1490474 | Up | chr11 | Upstream | PRG2 |
| T081968 | 0.025950723 | 2.2097847 | Up | chr12 | Downstream | RAB3IP |
| T081998 | 0.027389356 | 2.6591017 | Up | chr12 | Upstream | RAB3IP |
| T085524 | 6.00882E-05 | 3.9980095 | Up | chr12 | Downstream | TXNRD1 |
| T086224 | 0.022891761 | 4.0423404 | Down | chr12 | Upstream | ANKRD13A |
| T099792 | 0.001183757 | 3.8339577 | Up | chr13 | Downstream | TFDP1 |
| T101828 | 0.020234554 | 2.2719188 | Down | chr14 | Downstream | NFATC4 |
| T107139 | 0.014378521 | 3.0668982 | Up | chr14 | Upstream | DNAL1 |
| T112710 | 0.004366469 | 3.2371822 | Up | chr15 | Upstream | NIPA2 |
| T119232 | 0.015689163 | 2.0786343 | Up | chr15 | Downstream | NEO1 |
| T131413 | 0.040198641 | 2.4049612 | Up | chr16 | Upstream | BC068290 |
| T153074 | 0.01281594 | 2.0493837 | Down | chr17 | Upstream | WIPI1 |
| T167504 | 0.001883379 | 3.4387215 | Up | chr19 | Upstream | CERS4 |
| T182488 | 0.022842462 | 2.3834708 | Up | chr19 | Downstream | A1BG |
| T204141 | 0.008947259 | 2.071424 | Down | chr2 | Upstream | NUP35 |
| T227812 | 0.003140257 | 2.0782057 | Down | chr21 | Downstream | ITGB2 |
| T228063 | 0.001130167 | 4.512252 | Up | chr21 | Upstream | SLC19A1 |
| T231721 | 0.000294846 | 4.5599376 | Up | chr22 | Upstream | KREMEN1 |
| T250130 | 0.021961405 | 5.5068656 | Up | chr3 | Upstream | RPN1 |
| T275597 | 2.4436E-07 | 42.1565429 | Up | chr4 | Downstream | IRF2 |
| T284116 | 0.002575894 | 2.0151746 | Up | chr5 | Upstream | TMEM171 |
| T304390 | 0.002587025 | 3.0637387 | Up | chr6 | Upstream | HSP90AB1 |
| T335854 | 2.09683E-06 | 5.0807059 | Up | chr7 | Downstream | ABCB8 |
| T353439 | 0.000628621 | 3.985983 | Up | chr8 | Upstream | TOP1MT |
| T355493 | 0.000229031 | 2.9047396 | Up | chr9 | Upstream | C9orf123 |
| TCONS_00014980 | 0.047128035 | 2.7085507 | Up | chr8 | Upstream | ADAM9 |
| TCONS_00020595 | 5.78561E-07 | 117.4889202 | Up | chr12 | Downstream | CAMKK2 |
| uc021ruo.1 | 0.007621346 | 2.8502196 | Up | chr14 | Downstream | SPTB |

Seqname: the sequence identifier of lncRNA; nearby gene: the accession number of nearby coding gene with the lncRNA.
